# The Role of Glycolysis in Tumorigenesis—The Many Unresolved Issues

**DOI:** 10.3390/cells15060499

**Published:** 2026-03-11

**Authors:** Fabrizio Marcucci, Shibo Wei, Marco Cordani

**Affiliations:** 1 Department of Pharmacological and Biomolecular Sciences, University of Milan, via Trentacoste 2, 20134 Milan, Italy; 2 Department of Precision Medicine, School of Medicine, Sungkyunkwan University (SKKU), Suwon 16419, Republic of Korea; weishibo@g.skku.edu; 3 Department of Biochemistry and Molecular Biology, Faculty of Biological Sciences, Complutense University of Madrid, 28040 Madrid, Spain; mcordani@ucm.es; 4 Instituto de Investigación Sanitaria San Carlos (IdISSC), 28040 Madrid, Spain

**Keywords:** glycolysis, tumorigenesis, lactate, moonlighting, danger, stress

## Abstract

The upregulation of glycolysis and resultant lactate production (hereafter referred to as fermentative glycolysis) even under normoxic conditions has been considered a hallmark of cancer. In recent years, however, it has become clear that fermentative glycolysis in tumors is not as all-inclusive as originally thought. Nevertheless, many tumor types at different stages of progression are characterized by a predominantly glycolytic metabolism. Fermentative glycolysis in tumors supports several different functions: energy production in the form of adenosine triphosphate molecules, the maintenance and amplification of glycolytic metabolism itself, the feeding of oxidative metabolism through the production of lactate, the generation of metabolic intermediates for biomass production, and the execution of non-metabolic, non-canonical, so-called moonlighting functions. This knowledge, however, raises a number of different questions which, by and large, are still unanswered today. Are there different degrees of glycolysis upregulation in order to support the different functions? How is fermentative glycolysis maintained even under normoxic conditions? Why do moonlighting functions exist, given that they are unrelated to the metabolic steps of glycolysis? Moonlighting functions are generally discussed in the context of tumorigenesis, but do they exist also in non-transformed cells? Do they occur in a coordinated manner in all tumor cells or are they activated selectively depending on the tumor type, tumor stage, or on the inducing stimulus? While these issues are mostly unresolved, in this article we propose some tentative answers which, we hope, may promote new research directions which may further our understanding in this field.

## 1. Introduction

Glycolysis converts glucose to pyruvate with a net yield of 2 adenosine triphosphate (ATP) molecules per glucose molecule. Under aerobic conditions, pyruvate then enters oxidative metabolism [tricarboxylic acid (TCA) cycle and oxidative phosphorylation (OXPHOS)] upon conversion to acetyl-coenzyme A (CoA), which is then metabolized to CO_2_, reduced nicotinamide adenine dinucleotide (NADH), and reduced flavin adenine dinucleotide. In OXPHOS, these molecules are further metabolized to generate ATP (up to 34 molecules of ATP per glucose molecule) [1]. Glucose is not the only molecule that fuels the TCA cycle. Fatty acids can also feed the TCA cycle upon β-oxidation to acetyl-CoA, which also occurs with glutamine upon conversion to α-ketoglutarate. In anaerobiosis, i.e., in the absence of oxygen, pyruvate does not enter the TCA cycle, but is reduced (fermented) to lactate in a reaction catalyzed by lactate dehydrogenase (LDH). With the term fermentative glycolysis, we refer to the metabolization of glucose to pyruvate (glycolysis in the real sense of the word) plus the reduction in the pyruvate to lactate. The balance between glycolysis and oxidative metabolism is tightly regulated, and this balance may become dysregulated in cancer [2].

**Figure 1:**
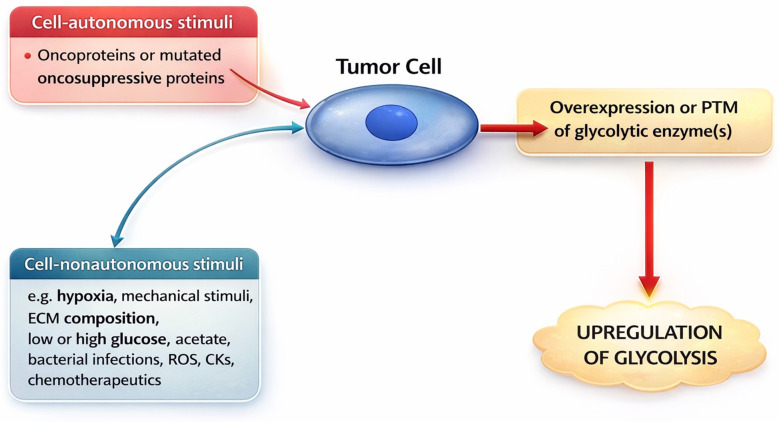
Stimuli that upregulate fermentative glycolysis. Cell-autonomous or -nonautonomous stimuli can induce overexpression or PTM of glycolytic enzyme(s). These, in turn, promote overall upregulation of fermentative glycolysis with increased production of metabolites, in particular lactate, which can induce several biological effects. PTM, post-translational modification; ECM, extracellular matrix; ROS, reactive oxygen species; CK, cytokine. The figure was created with ChatGPT OpenAI version 5.4.

About one century ago, Otto Warburg [3] discovered that tumor cells can undergo reprogramming from oxidative metabolism to fermentative glycolysis even under aerobic conditions. This phenomenon has been recognized as one of the hallmarks of cancer [4]. Subsequent studies found that fermentative glycolysis occurs in response to a large variety of stimuli, in addition to hypoxia [5]. These stimuli can be cell-autonomous or -nonautonomous (Figure 1).

Cell-autonomous stimuli that upregulate fermentative glycolysis in tumor cells are, for example, oncoproteins [6,7]. Thus, oncoproteins which have been shown to upregulate glycolysis include Kristen rat sarcoma virus [8], c-Myc [7], Src [9], EWS-FL1 [10], oncogenic viruses [11], the phosphoinositide 3-kinase/AKT/mechanistic target of rapamycin (mTOR) pathway [12], Wnt/β-catenin [13], epidermal growth factor receptor [14], and human epidermal growth factor receptor 2 [15]. Oncosuppressive proteins can also upregulate glycolysis if they lose their oncosuppressive function and/or acquire oncogenic functions [16]. Some of these oncoproteins (e.g., c-Myc) promote fermentative glycolysis by acting as transcription factors (TFs) which increase the expression of one or more glycolytic enzymes [7,8,10].

As regards cell-nonautonomous stimuli that upregulate fermentative glycolysis, the first to be mentioned is hypoxia, the prototypic inducer of fermentative (anaerobic) glycolysis [17]. In addition, mechanical stimuli (e.g., compressive stress), increased hyaluronan concentration in the extracellular matrix [18], too low or too high glucose levels in the extracellular milieu [19,20], extracellular acetate [21], bacterial infections [22], antitumor therapeutics [23], reactive oxygen species (ROS) [24], or cytokines [25] can upregulate fermentative glycolysis. Importantly, cell-autonomous and -nonautonomous stimuli can cooperate in inducing metabolic reprogramming towards glycolysis [2].

It has also been proposed that fermentative glycolysis may become predominant over oxidative metabolism, because the demand for oxidized nicotinamide adenine dinucleotide (NAD^+^) required to support oxidation reactions exceeds the rate of ATP turnover [26]. A mechanism of this kind may complement cell-autonomous or -nonautonomous stimuli that lead to an upregulation in glycolysis. Available NAD^+^ would then become limiting, and this would promote fermentative glycolysis despite the available oxygen.

Upregulation of glycolysis in response to these stimuli has two consequences. First, fermentative glycolysis becomes the predominant energy-producing pathway. Second, tumor glycolysis becomes upregulated compared to normal tissues [27]. Upregulation of glycolysis is the result of quantitative (e.g., overexpression of glucose transporters or glycolytic enzymes or overproduction of metabolites) and/or qualitative (e.g., PTMs or expression of embryonic isoforms of glycolytic enzymes) changes in glycolytic enzymes [28]. When such changes apply, initially, to an individual enzyme, this leads to an overall upregulation of glycolysis [28,29,30]. Upregulated glycolysis can be transmitted to nearby cells by means of intercellular communications [31].

While fermentative glycolysis may become the main energy-producing pathway in tumors, this does not hold true for all tumors [32] and, importantly, even in tumors with a predominant glycolytic metabolism, oxidative metabolism is never completely inhibited. Thus, in all tumor cell lines that were investigated, fermentative glycolysis and oxidative metabolism coexisted, albeit at varying percentages depending on the tumor type and stage [33,34]. This metabolic heterogeneity has been confirmed in vivo, in non-small cell lung cancer patients who had been intraoperatively infused with ^13^C-glucose [27]. In fact, it has been claimed that the coexistence of glycolytic and oxidative metabolism is required to support full tumorigenicity [35]. This has been demonstrated for different tumor types [36,37].

## 2. The Outputs of Fermentative Glycolysis

**Figure 2:**
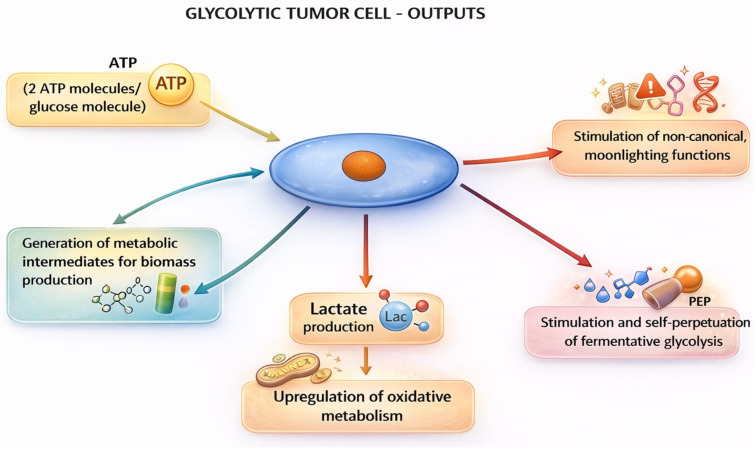
Outputs of upregulated fermentative glycolysis in tumor cells. The figure depicts the outputs of fermentative glycolysis that have been induced/upregulated in response to cell-autonomous or -nonautonomous stimuli. ATP, adenosine triphosphate. The figure was created with ChatGPT OpenAI version 5.4.

After having considered the circumstances that lead to the upregulation of glycolysis in tumor cells, allowing it to become the predominant energy-producing pathway, one is led to ask which outputs of fermentative glycolysis influence tumor cell proliferation and tumor progression (Figure 2).

The first output is, obviously, the production of energy in the form of ATP molecules. We have already discussed that oxidative metabolism is much more efficient in producing ATP than fermentative glycolysis. In fact, it has been shown that oxidative metabolism generates > 90% of the overall ATP asset in aerobic conditions [38]. So, why do many but not all tumor cells recur to glycolysis if ATP production is a crucial item for cell survival and/or proliferation? One advantage of glycolytic ATP production is that ATP is produced at a faster rate than in oxidative metabolism [39,40]. In accordance with this view, an earlier study showed that ATP consumption can increase glycolytic flux [41]. Moreover, it has been reported that tumor cells relying predominantly on glycolysis have a growth advantage compared to similar cells relying predominantly on oxidative metabolism [42]. In fact, the rapid onset of ATP production may be of particular utility for proliferating cells, since it has been shown that fermentative glycolysis is mainly used in the S phase of the cell cycle [43,44]. In fact, most, albeit not all, tumor cells undergo rapid proliferation. Rapid proliferation, however, is not an exclusive characteristic of tumor cells and, in fact, upregulation of fermentative glycolysis has also been shown to occur in non-transformed cells undergoing rapid proliferation, like fibroblasts [45] or mitogen-stimulated lymphocytes [46].

The second reason as to why tumor cells may reprogram metabolism towards glycolysis is the production of metabolic intermediates that facilitate tumor cell proliferation and tumor growth. Thus, glycolysis upregulation in tumor cells can feed branching metabolic pathways to support the synthesis of nucleotides, lipids and amino acids that are needed to support tumor cell proliferation [47]. In triple-negative breast cancer tissues, it has been shown that glucose directly feeds ribose phosphate, amino acid synthesis, lactate, and the TCA cycle [48]. These results suggest that both the oxidative and fermentative metabolism of glucose collaborate in generating the intermediates required to support the biological machinery of tumor cells.

In support of this possibility and, somehow paradoxically given the reprogramming towards glycolysis, is that one consequence of the upregulation of fermentative glycolysis is the feeding of oxidative metabolism. In fact, the final metabolite of fermentative glycolysis, lactate, and the ensuing lactic acidosis, are now recognized as a major fuel and stimulus for the upregulation of oxidative metabolism, respectively [49]. Once extruded, lactate can reenter cells through the monocarboxylate transporter (MCT) 4, and it is oxidized inside the cytoplasm to pyruvate, which then enters the TCA cycle upon conversion to acetyl-CoA [50]. Lactate, however, also upregulates OXPHOS through several other mechanisms, including the stimulation of mitochondrial biogenesis [51] and stimulation of the electron transport chain (ETC) upon entering the mitochondrial matrix [52]. On the other hand, it has also been reported that lactylation of some mitochondrial proteins leads to their inactivation and, consequently, to the inhibition of OXPHOS because of the reduced generation of acetyl-CoA [53]. Finally, acidosis, the accompanying feature of lactate release, has also been shown to stimulate oxidative metabolism [54,55,56]. These observations reinforce the knowledge that cancer metabolism strives towards heterogeneity: when one energy-producing pathway predominates, mechanisms are at work from a certain point on that aim at reestablishing a more balanced equilibrium between the two pathways [2,32].

In contrast with the previous effect, upregulated glycolysis also puts in place self-perpetuating mechanisms, i.e., it promotes the upkeeping of itself. Thus, glycolytic enzymes have been shown to mediate glycolysis upregulation in response to cell-autonomous or -nonautonomous stimuli [57,58] through a variety of different mechanisms, e.g., [30,59,60].

A further output of upregulated glycolysis are non-canonical, so-called moonlighting functions. These functions are mediated by glycolytic enzymes, either overexpressed or modified, or glycolytic metabolites such as lactate or pyruvate. Over the years, a bewildering number of such functions have been reported in the literature. It would go beyond the scope of this article to list them all. Several recent reviews have described and summarized these functions [32,61,62,63]. Here, we briefly list the main classes of these functions [5,64]: induction of tumor-driving mutations, increased expression and/or activity of oncoproteins, epigenetic modifications including acetylation or lactylation of histone proteins, increased tumor cell proliferation and/or senescence, promotion of DNA damage response (DDR), antiapoptotic effects, induction of autophagy, and reduction in drug accumulation and immunosuppressive effects.

## 3. The Molecular Changes That Underlie the Acquisition of Moonlighting Functions

Moonlighting functions appear in tumor cells as a result of the upregulation of glycolysis. This leads us to ask which molecular changes underlie the acquisition of these functions. One consequence of the modifications that will be discussed in the following is that glycolytic enzymes change their usual cytoplasmic localization. In many cases, the modifications lead to the localization of glycolytic enzymes to the nucleus, where they exert non-canonical, moonlighting functions.

**Figure 3:**
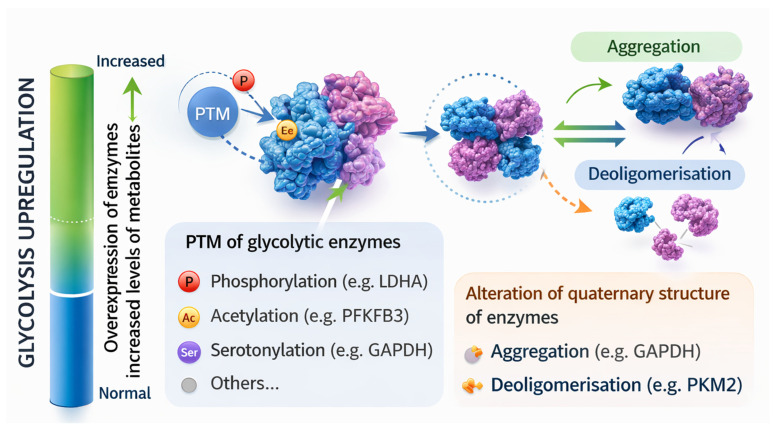
Mechanisms underlying moonlighting functions of glycolytic enzymes or metabolites. Moonlighting functions may become detectable following overexpression of glycolytic enzymes, which may induce overall upregulation of fermentative glycolysis and increased levels of glycolytic metabolites (e.g., lactate); PTM of glycolytic enzymes; alteration of the quaternary structure of glycolytic enzymes leading to their aggregation or deoligomerisation (e.g., tetramer–dimer/monomer conversion of PKM2). GAPDH, glyceraldehyde-3-phosphate dehydrogenase; LDHA, lactate dehydrogenase; PFKFB3, 6-phosphofructo-2-kinase/fructose-2,6-biphosphatase 3; PKM2, pyruvate kinase isoform M2; PTM, posttranslational modification. The figure was created with ChatGPT OpenAI version 5.4.

Moonlighting functions of glycolytic enzymes may become detectable in tumor cells due to the increased expression of an individual glycolytic enzyme that will be the effector of this function (Figure 3). Even if this function is carried by an individual enzyme, this may occur in the context of an overall upregulation of glycolysis, e.g., [65,66,67].

In other cases, moonlighting functions are the result of PTMs of a glycolytic enzyme occurring in response to the stimuli that have been discussed in previous sections. These PTMs include phosphorylation, e.g., [68,69], acetylation [70,71], serotonylation [72], citrullination [30], O-GlcNAcylation [73], and nitrosylation [74].

Yet another possibility, non-exclusive with the previous ones, is represented by modifications of the quaternary structure of glycolytic enzymes. Thus, glyceraldehyde-3-phosphate dehydrogenase (GAPDH) has been shown to undergo aggregation in response to oxidative stress, a change that leads to the opening of permeability transition pores and subsequent mitochondrial dysfunction [75]. Pyruvate kinase isoform M2 (PKM2) is a PK isoform that exists as a highly active tetramer or low-activity monomeric/dimeric forms. The latter forms localize to the cell nucleus where they perform non-canonical functions [76,77]. Nuclear localization of PKM2 is the consequence of the exposure of nuclear localization signals (NLS) upon tetramer–monomer/dimer conversion [78]. Interestingly, tetramer–monomer/dimer conversion of PKM2 has been suggested to also endow it with protein kinase activity, which is absent in its tetrameric form [79,80]. An interesting example for nuclear localization of PKM2 upon tetramer–dimer/monomer conversion has been reported in a non-tumor system, i.e., lipopolysaccharide-stimulated dendritic cells [81]. Upon stimulation, PKM2 was acetylated at Lys^433^. This modification destabilized PKM2 and promoted its nuclear localization. Here, PKM2 was associated with c-Rel and an increased expression of *Il12p35*, which, in turn, facilitated T helper (Th) 1 cell differentiation.

Nuclear localization, however, can also result from overexpression [82] or PTM of a glycolytic enzyme [83,84]. Nuclear localization of the glycolytic enzyme Aldolase A, for example, has been shown to be involved in the repair of DNA double-strand breaks upon nuclear localization [85]. In addition to nuclear localization, mitochondrial localization of glycolytic enzymes has also been reported [86]. Thus, mitochondrial phosphoglycerate kinase 1 phosphorylated and activated pyruvate dehydrogenase kinase 1, thereby reducing pyruvate utilization in the TCA cycle and increasing fermentative glycolysis and lactate production [86]. This represents a feed-forward mechanism whereby a glycolytic enzyme amplifies glycolysis through a non-canonical mechanism in a non-canonical localization. PKM2 has also been shown to translocate to mitochondria in response to oxidative stress [87]. Here, PKM2 interacted with and phosphorylated the antiapoptotic protein B-cell lymphoma 2 (Bcl2), thereby preventing the degradation of Bcl2 and inhibiting apoptosis.

While most attention has been focused on the moonlighting functions of glycolytic enzymes [88], glycolytic metabolites can also exert non-canonical functions, unrelated to their role in metabolism. One example is the non-canonical functions of pyruvate. Pyruvate enters the TCA cycle upon transformation to acetyl-CoA or, anaplerotically, upon transformation to oxalacetate or upon transamination. A non-canonical function of pyruvate was suggested by its inhibitory effect on the anti-neoplastic activity of carnosine on glioblastoma multiforme (GBM) cells even when oxidative ATP production was blocked [89]. In fact, pyruvate may exert antioxidant effects, as shown by the protection afforded to human neuroblastoma cells from hydrogen peroxide toxicity [90]. These effects were the consequence of scavenging H_2_O_2_-induced ROS, suppressing superoxide production by submitochondrial particles, and reducing the oxidative stress-induced decrease in mitochondrial membrane potential. More recently, it has been shown that increased mitochondrial pyruvate levels prevent growth in non-transformed cells by increasing the NADH/NAD^+^ ratio, which rewires metabolism towards gluconeogenesis and suppresses glycolysis [91]. Decreased amino acid synthesis caused reduced protein synthesis and inhibited cell growth. The precursor of pyruvate, phosphoenolpyruvate (PEP), was also found to perform non-canonical functions [92]. A glucose-poor tumor microenvironment (TME) decreased glycolytic metabolism in tumor-infiltrating T lymphocytes, thereby limiting the tumoricidal function of these cells. Such inhibition of T cell effector functions was due to reduced levels of PEP in T cells. Overexpression in these cells of PEP carboxykinase 1, which converts oxalacetate into PEP, stimulated the effector functions of T cells, thereby prolonging the survival of melanoma-bearing mice. Additionally, PEP has been shown to inhibit the differentiation of non-transformed Th17 cells through binding to the TF JunB [93]. This caused a DNA-binding inhibition in the JunB/basic leucine zipper transcription factor ATF-like (BATF)/interferon regulatory factor 4 (IRF4) complex, thereby regulating the transcriptional program of Th17 cells.

Lactate, the final metabolite of fermentative glycolysis, and its acid, have garnered great interest in recent years. Lactate has long been considered a waste product of fermentative glycolysis. As already discussed (Section 2), lactate can serve as a fuel for oxidative metabolism [49,94,95,96]. In addition, lactate has several non-canonical activities. Beforehand, it should be noted that lactate can enter or exit cells through MCTs: MCT1 is used mainly for cell exit and MCT4 for cell entry [97]. In addition, extracellular lactate can bind to the proton-sensitive cell surface receptors G-protein-coupled receptor (GPR) 81/hydrocarboxylic acid receptor 1 and GPR132 [98]. A prominent non-canonical activity of lactate that has been described in recent years is the lactylation of proteins, both histone and non-histone proteins. The plethora of biological activities, including tumor-promoting effects [99], that are modulated in response to this PTM in cancer and non-cancer settings have been reviewed [50,100,101]. As regards the other non-canonical activities of lactate, it would go beyond the scope of this article to enter into a detailed discussion about them. Nevertheless, we will briefly mention some of the most relevant ones. Thus, lactate and lactate acidosis exert immunosuppressive effects that support tumor growth and dissemination through different mechanisms of action, e.g., [102,103,104]. Moreover, lactate has been shown to regulate the cell cycle [105], to promote resistance to different antitumor drugs, e.g., [106,107], to accelerate tumor growth [108], and to induce EMT [109,110]. Interestingly, a link between histone lactylation and immunosuppressive activity has been recently described [111], as it was shown that histone lactylation induced a transcriptional program in microglia/macrophages in GBM that led to the upregulation of CD47, a cell-surface molecule that acts as a “don’t eat me” signal that suppresses phagocytosis.

## 4. Several Unanswered Questions About the Upregulation and Function of Fermentative Glycolysis in Tumor Cells

The previous sections have briefly summarized the fundamentals of fermentative glycolysis in tumors. Particular attention has been devoted to some new insights concerning the non-canonical functions of glycolytic enzymes and metabolites. Yet, current knowledge raises several questions on tumor glycolysis that are still unanswered. In the following, some of these questions will be addressed, and some tentative answers proposed, that will require experimental verification.

### 4.1. How Much Glycolysis to Do What?

As already discussed, rapidly proliferating cells, either normal or tumor cells, recur to fermentative glycolysis in order to satisfy several metabolic requirements. In addition, a large number of cell-autonomous and -nonautonomous stimuli can also upregulate glycolysis. This raises the question whether constitutive metabolic needs, on one hand, and stimuli, on the other hand, give rise to additive or synergistic upregulation of glycolysis, or if each one induces a maximal upregulation that cannot be further increased. Another possibility, non-exclusive with the previous ones, is that said stimuli select tumor cells that have an upregulated baseline level of glycolysis compared to non-transformed cells. Evidence for this latter possibility has been proposed [20]. In any case, one is led to ask whether there is a correlation between the level of upregulated glycolysis and one or more of the outputs that have been discussed before. Thus, it might be speculated that a minimum level of upregulated glycolysis might be required to support metabolic needs (e.g., rapid proliferation), but a higher level might be necessary to perform moonlighting functions. In fact, such a possibility is supported by the knowledge that, whenever intentionally investigated, moonlighting functions were found to occur in tumor cells that overexpress glycolytic enzymes compared to normal cells.

### 4.2. How Is Fermentative Glycolysis, i.e., the Warburg Effect, Maintained Under Normoxic Conditions?

The reasons as to why fermentative glycolysis is upregulated in tumor cells have been discussed in a previous section. Many of the stimuli that upregulate glycolysis are self-limiting, i.e., they vanish after a certain period of time. If these stimuli disappear, then how is fermentative glycolysis preserved once the stimulus is no longer present? This is the case, for example, when tumor cells upregulate glycolysis in the presence of anaerobiosis, and continue to rely, predominantly, on fermentative glycolysis once they return to aerobic conditions. We suggest that in this and similar cases, epigenetic changes induced by self-limiting stimuli can be transmitted to the cellular offspring for a prolonged, undefined period of time.

Several observations support this view. Thus, in non-transformed cells (C2C12 mouse skeletal myoblasts) glycolysis was upregulated following low-temperature stress (35 °C) [112]. This was paralleled by epigenetic changes, including expression of the methyltransferase Dnmt1 as well as the histone acetyltransferase Gcn5. Other epigenetic changes have been shown to upregulate, either directly or indirectly, the expression of genes encoding glycolytic enzymes [113,114]. Epigenetic changes of this kind can be inherited through different mechanisms [115]. This suggests that one mechanism allowing for the establishment of the Warburg effect in normoxic tumor cells is the inheritance of epigenetic changes in response to the cell-autonomous or -nonautonomous stimuli that have been discussed in previous sections. Interestingly, lactate itself, through lactylation of histone proteins and consequent activation of the transcription of genes encoding glycolytic enzymes, has been shown to be an epigenetic regulator of glycolysis in a non-tumor system [116]. In this regard, it is interesting to note that lactylation of histone H3, mainly at distal regulatory regions, characterizes trained monocytes/macrophages, which respond with enhanced production of cytokines to a secondary stimulation [117]. Thus, fermentative glycolysis itself, through lactate production and consequent lactylation, may also be at the origin of epigenetic changes which promote self-maintenance of glycolysis.

### 4.3. The Mystery of the Moonlighting Functions—The Many Unresolved Issues

As already mentioned, the number of non-canonical functions of glycolytic enzymes and metabolites, i.e., functions unrelated to their role in metabolism, is very large, to the extent that it is becoming difficult to list them in a single, even if extensive, article. Not surprisingly, then, they raise many questions.

Thus, when considering the moonlighting functions as a whole, one is led to ask whether they are induced coordinately altogether, or in subgroups, or individually in response to some stimuli but not others, and/or if their induction depends on the responding cell. Another question is whether these functions are induced only in tumor cells or whether they are also induced in non-transformed cells in response to similar stimuli. Last but not least, one would like to know the reason as to why these functions are activated in tumor cells. At present, it is only possible to give some preliminary answers to these questions and formulate hypotheses that await experimental verification.

One variable that may influence whether moonlighting functions are induced altogether, in subgroups, or individually is the duration and intensity of the stimulus that upregulates glycolysis. Thus, a relatively weak or short stimulus may induce one or a few of these functions, while a stronger stimulus or one of longer duration may induce a larger number of functions. The occurrence of graded responses to stimuli of varying duration and intensity has already been demonstrated in other biological systems [118,119]. The other variable that may influence the spectrum of moonlighting functions is the responder cell. Cells of different tumor types or tumor stages may respond differently to the same stimuli. These are testable hypotheses that warrant experimental investigation in order to gain a more complete view of the breadth and significance of glycolytic moonlighting functions in tumorigenesis.

Concerning whether glycolysis-associated moonlighting functions also occur in normal, non-transformed cells, the answer is yes [120,121,122], although much less information is available than for tumor cells. Several examples of moonlighting functions occurring in non-transformed cells will be discussed in the remainder this article. As we have discussed in the previous section, glycolysis-promoted epigenetic modifications can also occur in non-transformed cells [112]. Moreover, and most importantly, upregulated glycolysis can induce tumor-initiating events by promoting, among others, epigenetic changes, reviewed in [64]. On the other hand, the role of epigenetic modifications in facilitating neoplastic transformation and tumorigenesis is now firmly established [123,124,125]. Altogether, while no studies have been performed to investigate whether a stimulus that upregulates glycolysis induces inheritable epigenetic changes in non-transformed cells, the ensemble of the results that have been discussed here makes this a likely possibility that deserves investigation, especially given its possible role in inducing tumor-initiating events.

**Figure 4:**
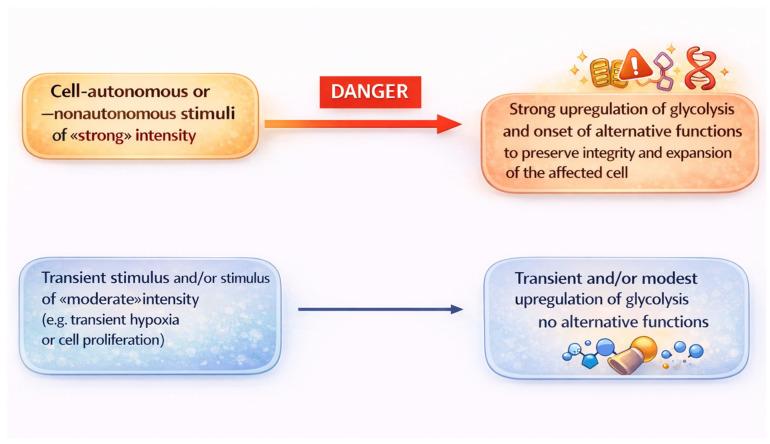
Stimuli inducing upregulation of glycolysis. Some stimuli of moderate intensity induce a transient and/or modest increase in fermentative glycolysis, which responds to the metabolic demands of the cell. Other stimuli of strong intensity, whether cell-autonomous or -nonautonomous, are perceived as danger signals by the affected cell and lead to strong upregulation of fermentative glycolysis with the onset of moonlighting functions. The figure was created with ChatGPT OpenAI.

One of the most difficult questions to address is why moonlighting functions are induced in tumor cells but, as we have just discussed, also in non-transformed cells. Stated differently, why do glycolytic enzymes and metabolites mediate functions that are totally unrelated to their role in glycolytic metabolism, whether in transformed or non-transformed cells? In spite of the scarcity of data on this point, we can detail some speculations that can be used as working hypotheses for future research. First of all, it is striking to note that most moonlighting functions promote the survival and expansion of the affected cell(s). This is the case for increased proliferation, promotion of DDR, antiapoptotic effects, and the reduction in drug accumulation. The induction of immunosuppressive effects also protects tumor cells, since they shield them from dangerous and potentially lethal immune responses. We have already proposed that glycolysis-associated drug resistance may represent a response of transformed or non-transformed cells to stimuli which, in principle, could undermine the integrity of the target cell(s) [28]. Herein, we propose that moonlighting functions, altogether, represent protective responses to potentially dangerous signals (Figure 4). Historically, the term danger signal was coined to define stimuli that induce innate immune responses that are initiated upon binding to pathogen-associated pattern-recognition receptors (PRR) [126,127]. Later, it was found that endogenous molecules, whether or not released from injured cells, are also able to interact with PRRs and non-PRRs in innate immune cells, thereby inducing sterile inflammatory responses aimed at repairing injured tissues [128]. Similarly, cell-autonomous or -nonautonomous stimuli induce upregulation of fermentative glycolysis and moonlighting functions. But which are the properties that endow these stimuli to be perceived as danger signals? We propose that this occurs when they exceed a given threshold of intensity. In this case, fermentative glycolysis is “overactivated” and one or more of the following changes occur. First, an increased expression of a glycolytic enzyme until a critical concentration is achieved, which allows it to interact with a non-canonical target. Second, the induction of a PTM of a glycolytic enzyme increases its affinity for a non-canonical target. Third, the induction of a modification of the quaternary structure of a glycolytic enzyme allows the enzyme to reach a non-canonical location (e.g., nucleus of mitochondria), where it performs its moonlighting function.

The crucial point concerning the model that we propose is that the affinity of the interaction between a given glycolytic enzyme for its substrate would be higher than the affinity of the interaction of the same enzyme (the ligand) with its non-canonical target. The latter interaction would become apparent only when the concentration and/or affinity of the ligand–target interaction exceed a given threshold in response to a dangerous or stressful stimulus.

But why are moonlighting functions associated with glycolytic metabolism? It is possible that the upregulation of glycolysis and appearance of moonlighting functions occur in response to the same signals, and this coincidence would have favored, energetically, the co-evolution of the two classes of functions, metabolic and moonlighting, within the same family of mediators.

It appears somehow paradoxical that a cellular response that is presumed to have protective effects towards the affected cell(s) supports tumorigenesis. This apparent paradox, however, is likely the result of the phenotype of the responding cells: in both non-transformed cells and transformed cells, moonlighting functions have protective effects, but in tumor cells, the responses to dangerous signals promote survival, thereby exacerbating the malignant properties.

So far, we have referred to moonlighting functions as the consequence of a supraphysiological increase in glycolysis in response to stimuli that we have referred to as danger signals. It can be appreciated, however, that moonlighting functions encompass several cellular stress responses (e.g., DDR, induction of autophagy, etc.) [129,130]. This suggests that moonlighting functions induced by danger signals represent an ensemble of cellular stress responses. The relationship and overlap between moonlighting functions and other cellular stress responses is a topic of obvious interest.

Yet another question is whether the protein domains responsible for the moonlighting functions are different from those responsible for the catalytic activity. On the basis of what we have discussed so far, the answer is yes. In fact, we have referred to the glycolytic enzyme PKM2, which is catalytically most active in its tetrameric form but performs moonlighting functions upon shifting to a dimeric/monomeric form and consequent exposure of an NLS, which allows it to shuttle into the nucleus, the site of its non-canonical activities [76,77]. In addition, however, one would like to know the exact domains of a given protein (e.g., a glycolytic enzyme) that performs the moonlighting function(s) and where it maps compared to the catalytic domain. Unfortunately, very little information is available about this important point. The exception is GAPDH, the glycolytic enzyme that has been most extensively investigated regarding this aspect. In fact, the knowledge acquired so far suggests that the domains that mediate the moonlighting functions are not overlapping [131] with those that mediate the catalytic activity, although a precise mapping of the different domains has, to our knowledge, not yet been reported. These observations suggest that the moonlighting functions of other glycolytic enzymes may also be mediated by protein domains different from those that are responsible for the catalytic activity.

## 5. Which Is the Most Important Output of Upregulated Glycolysis in Tumor Cells?

In a previous section, we summarized the most important outputs of upregulated glycolysis in tumor cells. A question that arises is which of these responses is the most crucial for tumor cell survival and expansion. In other words, is it possible to establish a hierarchy of relevance for tumorigenesis between the different outputs that have been discussed? This is not a question of mere academic interest, since an answer to it would allow us to predict which of the outputs we would like to inhibit in order to achieve maximal antitumor efficacy without affecting the integrity of normal cells.

A partial answer to this question derives from many observations that blocking one energy-producing pathway leads to a compensatory upregulation of the other pathway. Thus, the inhibitory effect of metformin on OXPHOS has been shown to lead to a compensatory upregulation of glycolysis [132,133]. In fact, this upregulation is at the origin of the most dangerous side effect of metformin, lactic acidosis [134]. A similar upregulation of glycolysis has been observed in response to the other ETC complex I inhibitor IACS-010759 [135]. Vice versa, upregulation of OXPHOS has been observed in response to the glycolysis inhibitor 2-deoxy-D-glucose [136]. Similarly, human lymphoma and colorectal carcinoma cells exposed to the MCT1 inhibitor AZD3965 upregulated oxidative metabolism [137]. These are just a few examples of the many that have been reported in the literature. Of note, while compensatory upregulation of the alternative energy-producing pathway has been reported in most cases, there are also exceptions. Thus, inhibition of OXPHOS was found to successfully eradicate quiescent leukemic stem cells without any evidence of glycolysis upregulation aimed at supporting survival of the cells [138]. These and similar results suggest the possibility of eradicating some tumor cell populations through the use of inhibitors of only one energy-producing pathway while in most cases it appears necessary to use inhibitors of both pathways in order to achieve maximal eradication of metabolically heterogenous tumor cells [139,140].

The crucial point to consider is that several metabolic pathways branch out of glycolytic and oxidative metabolism, and several metabolites of both pathways serve as precursors for biomass production. Thus, the pentose phosphate pathway (PPP) and glycogen synthesis pathway branch out of glycolysis, while glycerol, which is used for the synthesis of triglycerides and phospholipids, is produced from the glycolytic metabolite glyceraldehyde 3-phosphate. On the other hand, intermediates of the TCA cycle can serve as precursors of fatty acids and cholesterol which, in turn, can be used for the synthesis of steroid hormones, bile salts, and vitamin D. They can also serve as precursors of many non-essential amino acids, some of which, in turn, can serve as precursors of purines and pyrimidines. Porphyrins also derive the majority of their carbon atoms from TCA cycle intermediates. Finally, oxalacetate becomes a precursor of glucose in the gluconeogenesis pathway. As can be immediately appreciated, glycolytic and oxidative metabolism supply intermediates for essentially non-overlapping pathways. There are a few exceptions to this rule. Thus, many amino acids can be generated from precursors of oxidative and glycolytic metabolism, but in most, although not all cases (e.g., isoleucine), they differ between the two pathways. Another exception is reduced nicotinamide adenine dinucleotide phosphate, which can be generated not just during the oxidative phase of the PPP, but also by malic enzymes, by the TCA cycle enzymes isocitrate dehydrogenase 1 and 2, and by one-carbon metabolism [141]. Yet another exception is ATP itself, albeit it is produced, as already discussed, with greatly different efficiencies by the two pathways. The reasons as to why glycolytic ATP production by glycolysis may be preferred over that of oxidative metabolism in spite of the lower efficiency have been discussed in a previous section. On the whole, this knowledge suggests the possibility that ATP production is the most important metabolic output for tumorigenesis. On the other hand, other effects like the moonlighting functions or the generation of intermediates for biomass production may play a crucial role depending on the tumor type or tumor stage.

## 6. Conclusions

In this article, we have briefly summarized current knowledge on the role of fermentative glycolysis in tumorigenesis and, in what follows, we have raised several unanswered questions on this issue. Thus, we have discussed evidence suggesting that different degrees of glycolysis upregulation may give rise to different responses. We have also raised the problem regarding how fermentative glycolysis is maintained in aerobic conditions once the initiating stimulus has vanished, and have suggested that this may be related to inheritable epigenetic changes that accompany upregulation of glycolysis. The reason as to why upregulation of glycolysis is accompanied by non-canonical, moonlighting functions of glycolytic enzymes and metabolites is also an unresolved problem. Regarding this aspect, we have suggested that upregulation of glycolysis and the appearance of non-canonical functions may be responses to stimuli acting as danger/stress signals. These responses may be aimed at preserving the integrity and promoting the proliferation of the affected cell, which, in the case of tumor cells, would imply an increased tumorigenicity and malignancy. Finally, we have also asked which output of fermentative glycolysis is most important in promoting growth and dissemination. Evidence has been discussed which suggests that ATP production may be, indeed, the single most relevant effect in this regard and the one which may be the most desirable to inhibit from a therapeutic point of view. Many inhibitors of glycolysis and oxidative metabolism leading to decreased energy production have been described over the years but, so far, clinical trials that have been performed with these inhibitors have failed. It appears likely that, in order to obtain clinically relevant results, these compounds need to be targeted to the TME and tumor cells, whether as drug conjugates or nanoparticulate formulations [142,143]. As to the possibility of targeting moonlighting functions for therapeutic purposes, it seems necessary to obtain more information about their role in different tumor types and tumor stages, as well as biomarkers, before designing molecules able to interfere with these effects.

## Data Availability

No new data were created or analyzed in this study. Data sharing is not applicable to this article.

## Abbreviations

ATP, adenosine triphosphate; Bcl2, B-cell lymphoma 2; CoA, coenzyme A; DDR, DNA damage response; ETC, electron transport chain; GAPDH, glyceraldehyde-3-phosphate dehydrogenase; GBM, glioblastoma multiforme; GPR, G-protein-coupled receptor; LDH, lactate dehydrogenase; MCT, monocarboxylate transporter; NAD^+^, oxidized nicotinamide adenine dinucleotide; NADH, reduced nicotinamide adenine dinucleotide; NLS, nuclear localization signal; OXPHOS, oxidative phosphorylation; PEP, phosphoenolpyruvate; PKM2, Pyruvate kinase isoform M2; PPP, pentose phosphate pathway; PRR, pattern-recognition receptor; ROS, reactive oxygen species; TCA, tricarboxylic acid; TF, transcription factor; Th, T helper; TME, tumor microenvironment.
